# Astaxanthin-mediated Nrf2 activation ameliorates glucocorticoid-induced oxidative stress and mitochondrial dysfunction and impaired bone formation of glucocorticoid-induced osteonecrosis of the femoral head in rats

**DOI:** 10.1186/s13018-024-04775-z

**Published:** 2024-05-14

**Authors:** Weidan Wang, Hongyi Jiang, Jiachen Yu, Chao Lou, Jian Lin

**Affiliations:** 1 The Second Affiliated Hospital and Yuying Children’s Hospital of Wenzhou Medical University, Wenzhou, 325000 Zhejiang Province China; 2 Key Laboratory of Orthopedics of Zhejiang Province, Wenzhou, 325000, Zhejiang Province China; 3 The Second Clinical School of Medicine, Wenzhou Medical University, Wenzhou, 325000 Zhejiang Province China

**Keywords:** Glucocorticoid-induced osteonecrosis of the femoral head (GIONFH), Astaxanthin, Oxidative stress, Apoptosis, Nrf2 pathway

## Abstract

**Background:**

Osteonecrosis of the femoral head caused by glucocorticoids (GIONFH) is a significant issue resulting from prolonged or excessive clinical glucocorticoid use. Astaxanthin, an orange-red carotenoid present in marine organisms, has been the focus of this study to explore its impact and mechanism on osteoblast apoptosis induced by dexamethasone (Dex) and GIONFH.

**Methods:**

In this experiment, bioinformatic prediction, molecular docking and dynamics simulation, cytotoxicity assay, osteogenic differentiation, qRT-PCR analysis, terminal uridine nickend labeling (TUNEL) assay, determination of intracellular ROS, mitochondrial function assay, immunofluorescence, GIONFH rat model construction, micro-computed tomography (micro-CT) scans were performed.

**Results:**

Our research demonstrated that a low dose of astaxanthin was non-toxic to healthy osteoblasts and restored the osteogenic function of Dex-treated osteoblasts by reducing oxidative stress, mitochondrial dysfunction, and apoptosis. Furthermore, astaxanthin rescued the dysfunction in poor bone quality, bone metabolism and angiogenesis of GIONFH rats. The mechanism behind this involves astaxanthin counteracting Dex-induced osteogenic damage by activating the Nrf2 pathway.

**Conclusion:**

Astaxanthin shields osteoblasts from glucocorticoid-induced oxidative stress and mitochondrial dysfunction via Nrf2 pathway activation, making it a potential therapeutic agent for GIONFH treatment.

## Introduction

Glucocorticoids (GCs) are commonly used to treat various conditions such as acute spinal cord injury, viral infections, autoimmune diseases, and shock, owing to their potent immunosuppressive and anti-inflammatory effects. Nonetheless, in certain individuals, GC can lead to osteonecrosis of the femoral head (ONFH), with joint replacement typically regarded as a last-resort treatment option.

Numerous studies have explored the pathogenic mechanisms underlying GIONFH. So far, apoptosis, oxidative stress (OxS), osteogenesis dysfunction, vasoconstriction, lipid metabolism disorders, and coagulation and fibrinolysis system disorders have all been associated with GIONFH-related molecular mechanisms [1]. Recent research has shown that high doses of GC can induce osteoblast OxS [2]. In cases of excessive OxS, the buildup of reactive oxygen species (ROS) within cells leads to heightened endoplasmic reticulum (ER) stress, damage to mitochondrial DNA (mtDNA), impaired mitochondrial function, and ultimately, the activation of the Bcl-2/BAX apoptotic pathway [3]. Furthermore, the loss of mitochondrial membrane potential (MMP) has been connected to this process [4, 5]. In mouse osteoblast MC3T3-E1 cells, dexamethasone (Dex) stimulation causes proliferation to be maintained in the G1 phase, which in turn activates apoptosis [4]. Similarly, increased ROS expression has been found to induce ER stress and subsequently trigger apoptosis in primary osteocytes of C57BL/6 mice [6]. As a result, targeting the regulation of oxidative stress and apoptosis is crucial for addressing GIONFH.

Nuclear factor erythroid 2-related factor 2 (Nrf2), a vital transcription factor, is disrupted in various oxidative stress (OxS)-related diseases [7]. Heme oxygenase-1 (HO-1) and NADPH quinone oxidoreductase 1 (NQO1) are two examples of downstream genes activated by Nrf2 when it is released from its regulator, Keap1 [8]. Activators of the Nrf2 signaling pathway have been demonstrated to protect against several chronic diseases in experimental models, such as diabetic, autoimmune, respiratory, gastrointestinal, cardiac, and neurodegenerative diseases [8]. Nrf2 activation is effective in reducing OxS and apoptosis [9], highlighting its potential as a therapeutic target for GIONFH.

Astaxanthin (AST) is an orange-red, fat-soluble carotenoid found in lobsters and commonly used in aquaculture. It occurs in phytoplankton, yeast, microalgae, crustaceans, zooplanktons, and fish [10]. Notably, due to its anti-atherosclerotic, antioxidative, anti-aging, and anti-inflammatory properties, it has been proposed as a functional food and supplementary ingredient [11, 12]. AST has been reported to alleviate various inflammatory diseases, including inflammatory lung disease, rheumatoid arthritis, arteriosclerosis, gastritis, cerebral edema, and sepsis. There is also growing evidence highlighting the antioxidative properties of AST. Its effectiveness in reducing lipid peroxidation-induced damage by preserving membrane structure in a polyunsaturated fatty acid-rich membrane model has been demonstrated [13]. This effect is attributed to its polar end group extending into the polar region of the membrane bilayer [14]. Furthermore, due to its antioxidant and anti-inflammatory properties, AST’s potential role in the cardiovascular field is currently being explored [14]. In high-fat diet-induced hyperglycemic rats, AST reduced platelet aggregation and coagulation, increased fibrinolytic activity, and lowered antioxidant generation, endothelial cell protection, and lipid and lipoprotein levels [15]. The antioxidative effects of AST on the salivary glands in radiation-induced mouse models have also been shown [16]. Several studies have reported that AST activates the Nrf2 pathway [17, 18]. However, the therapeutic effect of AST on GIONFH remains unclear.

In general, our study demonstrated that the natural antioxidant AST may reduce oxidative stress and apoptosis for the treatment of GIONFH from the standpoint of regulating the oxidative stress and apoptosis of GIONFH. Additionally, we investigated the possibility that AST controlled oxidative stress and apoptosis via the Nrf2 signaling pathway. As a result, AST is a possible treatment for GIONFH.

## Materials and methods

### Reagents, media, and antibodies

Sigma Aldrich (St. Louis, MO, USA) and Macklin (Shanghai, China) provided the Dex and AST (≥ 96%), respectively. Penicillin/streptomycin, fetal bovine serum (FBS), and Dulbecco’s Modified Eagle’s Medium (DMEM) were purchased from Gibco BRL (Thermo Fisher Scientific, Waltham, MA, USA). Cell Signalling Technology (Denver, MA, USA) supplied antibodies against Bax, Bcl-2, cleaved caspase-9, Nrf2, glyceraldehyde-3-phosphate dehydrogenase, HO-1, cleaved caspase-3, and NQO-1. The other chemicals used in the study were of analytical quality and met the criteria for cell and tissue culture.

### Bioinformatic prediction

The chemical structural formula of AST active components was obtained using Pubchem database, and the chemical structural formula of TAX was mapped and its target was obtained using Targe Prediction database in Switzerland. The online database GeneCards was selected to identify GIONFH targets. Drug component-target disease models were established with Cytoscape software. In addition, protein-protein interactions (PPIs) were established with STRING software. Gene Ontology (GO) analysis and Kyoto Encyclopedia of Genes and Genomes (KEGG) enrichment analysis were performed with R.

### Molecular docking and dynamics simulation

Docking studies of compounds [AST, Protein Data Bank (PDB) ID: AXT] and targets (KEAP1, PDB ID: 6LRZ) were performed with AutoDock 4.2 (The Scripps Research Institute, LaJolla, San Diego, CA, USA). Chemical structures and biomolecules were preprocessed using PyMOL Molecular Graphics System version 2.6.0 (DeLano Scientific LLC, USA). Lamarckian genetic algorithm was used to search docking information. Critical residues in the binding pocket remain rigid during docking. The center of the target binding pocket was identified as the grid position. All runs passed 2.5 million rounds of energy evaluation steps. Representative pose selection was performed based on cluster analysis of docking poses.MD simulations of the docking complex were performed using charmm36 force field in GROMACS version 2020.3 package. The CGenFF server was used to generate the topology of the ligands. The protein-ligand system was embedded in an appropriately sized cubic box with periodic boundary conditions and CHARMM-modified TIP3P water model. The entire system was neutralized by adding eight Na + ions to the solution. The SHAKE algorithm was used to constrain all bond lengths involving hydrogen atoms. The particle mesh Ewald method was used to handle long-range electrostatic interactions within a 1.2 nm cut-off. The whole system was properly minimized, followed by NPT and NVT ensemble balancing steps. Apply 300 K and 1.0 bar temperature and pressure. Final production runs were performed using a Parrinello Rahman potentiostat to maintain constant temperature and pressure during the simulation. A 2 fs time step was applied.MD simulations of protein-ligand complexes were performed for 10 ns.

### Cell culture

Following successive trypsin-collagenase [19] digestion of the skulls of 1-day-old Sprague-Dawley (SD) rats, primary osteoblasts were extracted and grown in 5% CO2 at 37 °C in complete DMEM containing 1% (v/v) penicillin-streptomycin and 10% (v/v) FBS. The culture medium was changed every other day and cells were passaged until 80–90% confluence. Osteoblasts were subjected to treatment with 100 µM Dex at different time intervals, as per the clinical therapeutic dose and prior research, to establish a high-dose Dex setting [20, 21]. Cells underwent the following treatments: (1) Control group: for the same time as the Dex group, untreated osteoblasts were cultured. (2) Dex group: cells were cultured for an additional 48 h in complete DMEM after being treated with 100 µM Dex for 48 h; (3)Dex + AST group: cells were co-cultured with 1 and 5 µM AST for 48 h after being treated with 100 µM Dex for 48 h.

### Cytotoxicity assay

Cell counting kit-8 (CCK-8) assay (MedChemExpress LLC; Monmouth Junction, NJ, USA) was used to assess Dex and AST’s effects on osteoblast viability. Cells were seeded in 96-well plates at a density of 5 × 103 cells per well and subjected to treatment with varying doses of AST (0, 1, 5, 10, 20, 40, and 80 µM) for a duration of 48 and 72 h. After adding 10 µL CCK-8 reagent to each well, the cells were treated again for an hour. A Multiskan GO microdisk spectrophotometer (Thermo Fisher Science) measured optical density (OD) or absorbance at 450 nm.

### Osteogenic differentiation of primary calvarial osteoblasts

At a density of 5 × 104 cells per wall, we seeded cells into 24-well plates. According to the specified protocol, osteoblasts were cultured in DMEM with 10 mM β-glycosyl per phosphate and 20 mM ascorbic acid. Every other day, the media was replaced. An alkaline phosphatase (ALP) staining kit (Beyotime Institute of Biotechnology; Jiangsu, China) assessed ALP activity after 7 days of differentiation. Under osteogenic conditions, cells were cultured for 21 days before being fixed and stained with alizarin red S (Solarbio, Beijing, China) for mineralization and bone nodule formation.

### Western blotting

Using RIPA lysis buffer (Beyotime Institute of Biotechnology) with phosphatase and protease inhibitors (Sigma-Aldrich), total protein was extracted from cultured cells for 30 min at 4 ℃. Commercial kits were used for the purpose of isolating cytoplasmic and nuclear protein fractions following the manufacturer’s (Beyotime Institute of Biotechnology) instructions. As per the manufacturer’s instructions, after centrifuging cell lysates at 120,000 rpm for 30 min, the protein concentration in the supernatant was measured using a BCA protein assay kit (Beyotime Institute of Biotechnology). Protein (20 µg) from each sample was denatured by boiling for 5 min in sodium dodecyl sulfate (SDS)-loading buffer before being solubilized on SDS-polyacrylamide gels (10–15%). Protein strips were then transferred to polyvinylidene difluoride membranes (Merck Millipore; Burlington, MA, USA) for overnight incubation at 4℃. Following blocking with 5% skimmed milk diluted in Tris-buffered saline 0.1% Tween 20 (TBST) at room temperature for 2 h, blots were probed with the relevant primary antibodies for 12 h at 4℃. TBST was used three times to rinse the membranes before they were incubated with the secondary antibody, which was conjugated with HRP, for 4 h at RT. Enhanced chemiluminescence on a ChemiDoc XRS + imager (Bio-Rad; Hercules, CA, USA) was used to visualize the positive bands, and Image Lab V3.0 software (Bio-Rad) was used to quantify the densitometer results. The experiment was repeated three times.

### RNA extraction and qRT-PCR analysis

We purified total cellular RNA (tRNA) using TRIzol reagent, whereas Nanodrop measured RNA content using a 2000 spectrophotometer. Subsequently, cDNA was synthesized with RevertAid First Strand cDNA Synthesis Kit (Takara). Quantitative polymerase chain reaction (qPCR) was performed by using SYBR green detection reagent (Takara), LightCycler ® 96 real-time PCR system (Roche), IN, USA). Gene expression data were examined using the 2 ^− ΔΔCt^ method. Primer sequences are listed in Table 1.

**Table 1:** Sequences of all primers used in qPCR

Genes	Forward primer sequence (5’-3’)	Reverse primer sequence (5’-3’)
NOX1	TCGGAACTGCCTTGGCCTTG	AGGTGCCCCTCAGGAAGGAG
NOX2	GTGAGAGGCTGGTGCGGTTT	TCCCTCTGTCCAGTCGCCAA
NOX4	CGCGGATCACAGAAGGTCCC	TGGGCAGCTACATGCACACC
VEGFA	GAGCAGAAAGCCCATGAAGTG	ACTCCAGGGCTTCATCATTGC
HIF-1α	GCTGCCTCTTCGACAAGCTTA	TTGGTCTTCAGTTTCCGTGTCA
GAPDH	CAGGGCTGCCTTCTCTTGTG	GATGGTGATGGGTTTCCCGT

### Inhibition of the Nrf2 pathway

ML385 (Cell Signaling Technologies, MA, USA) is a highly selective Nrf2 inhibitor. Before Dex treatment, osteoblasts were pre-treated for 2 h with ML385 (10 µM). Cells were then collected for western blotting. The experiments were performed in triplicates.

### Terminal uridine nickend labeling (TUNEL) assay

TUNEL assay kit (Beyotime Shanghai, China) was used to detect apoptosis in cells and tissues. Osteoblasts cultured on glass coverslips were immersed for 30 min in 4% paraformaldehyde (PFA) followed by 10 min of incubation at 25 °C with 0.1% Triton X-100 and 3% H2O2, with phosphate-buffered saline (PBS) cleaning in between. Cells were routinely deparaffinized and rehydrated before being incubated for 20 min in proteinase K for paraffin sections. The samples were then exposed to a TUNEL detection solution (fluorescent label: terminal deoxynucleotidyl transferase, 9:1) for 1 h. Coverslips containing TUNEL-positive osteoblasts were sealed with DAPI-containing anti-fluorescence quenching and sealing solution before being visualized under a fluorescence microscope.

### Determination of intracellular ROS

The levels of intracellular ROS were measured with the aid of a Reactive Oxygen Species Assay Kit (Beyotime, Shanghai, China).

### Mitochondrial function assay

JC-1 staining was conducted to determine the MMP in osteoblasts from the control group, osteoblasts treated with Dex, and Dex + 5 µM AST as per the manufacturer’s instructions (Beyotime). Stained cells were examined under a fluorescent microscope (Olympus Life Science; Tokyo, Japan).

### Immunofluorescence

The primary skeleton cells were grown on glass, fixed with 4% PFA at room temperature for 15 min, and then incubated in PBS containing 0.5% (v/v) TritonX-100 for 20 min. Following an hour-long anti-incubation with 1% (w/v) goat seroproteins to prevent the binding of non-specific antibodies, the cells underwent an overnight anti-incubation with dissolved Bax (1:200) and BAX (1:200) at 4 °C. After washing PBS, color the cellular diaphragm for two hours with Alexa Fluor 488 or Fluor 594 labelled diopters (1:500). The cells were examined with an Olympus bx53 fluorescence microscope (Olympus Life Science) following a 5-minute DAPI reaction at room temperature. After that, examine the image with image J.

### GIONFH rat model construction

The Animal Ethics Committee of the Second Affiliated Hospital of Wenzhou Medical University and Yuying Children’s Hospital (Wenzhou, China) approved all animal experiments, which were conducted following the criteria described in the Guide for the Care and Use of Laboratory Animals (NIH, Bethesda, MD, USA). SD male rats (10 weeks old) were provided by the Shanghai Laboratory Animal Centre (SLAACAS; Shanghai, China) and housed under SPF conditions at 22–25 °C with a 12 h light/dark cycle. Standard rodent diet (Provimi KlibaAG, Kaiseraugst, Switzerland) consisted of 70-80% carbohydrates, 2.5% casein, 5% fat, 1% calcium, and 0.8% phosphorus; all animals had free access to clean water. After 1 week of acclimatization, the rats were randomly divided into control group (normal saline treatment), MPS model group and MPS + AST group (10 rats in each group). Briefly, 20 mg/kg/day methylprednisolone (MPS) (Pfizer, NY, USA) was injected intramuscularly into the right thigh of rats for 3 weeks during the first 3 days. Following MPS injection, AST (200 mg/kg/day) was administered by gavage to each rat. We randomly divided 30 rats into three groups (10 rats in each group): (1) control group (saline treatment), (2) MPS model (MPS treatment), and (3) MPS + AST (MPS and ast treatment) groups. Previous studies have reported that oral administration of 200 mg/kg AST resulted in higher AST half-life and first-extraction rates from the liver and gastrointestinal tract than intravenous injection. Therefore, we intragastrically administered 200 mg/kg/d AST to [22] rats. After 6 weeks, rats were sacrificed following an overdose anesthetic treatment regimen.

### Micro-computed tomography (micro-CT) scans

Micro-CT scanning was conducted to examine the femoral head’s structural details to gauge the extent of femoral head necrosis in rats. Experiments with micro-CT were conducted before tissue decalcification. On a micro-CT system (70 kV, 114 a; micro-CT 80 scanner; Scanco Medical, Bassersdorf, Switzerland), samples were scanned. To quantify the relative amount of bone in the proximal femur, trabecular number (Tb.N), thickness (TbTh.), and trabecular separation (Tb.Sp) were measured, along with bone volume/total volume (BV/TV).

### Histology, immunohistochemistry (IHC), and immunofluorescence

Fixed femurs were decalcified in 10% EDTA for four weeks, dehydrated using a gradient of ethanol (70–100%), cleaned with xylene, and then embedded in paraffin. Following the manufacturer’s directions, longitudinal 4-m-thick serial slices were stained with hematoxylin and eosin. As per the manufacturer’s protocol, anti-COL-1 and anti-HO-1 primary antibodies were incubated with 6-m-thick sections for IHC before being detected with a horseradish peroxide detection system (Vector Laboratories; Burlingame, CA, USA). ImageJ software was used to analyze the chromogenic intensity and immunohistochemical positivity.

### Enzyme-linked immunosorbent assay (ELISA)

The levels of rat serum of procollagen type I N-terminal propeptide (PINP, a marker of bone formation) and β-C-terminal telopeptide of type 1 collagen (β-CTX, a marker of bone resorption) were determined by ELISA method (Nanjing Jiancheng Bioengineering Institute, Nanjing, China).

### Statistical analysis

At least three different experiments were conducted. The GraphPad Prism software (San Diego, CA, USA) was used for all statistical analyses, and the data are presented as the mean ± standard error of the mean (SEM). Data analysis was conducted using the one-way analysis of variance, and group comparisons were down employing Tukey’s test. Means ± standard deviation is used to report experimental data, and statistical significance was defined as a p-value < 0.05.

## Results

### Bioinformatic analysis between astaxanthin and GIONFH

**Fig. 1:**
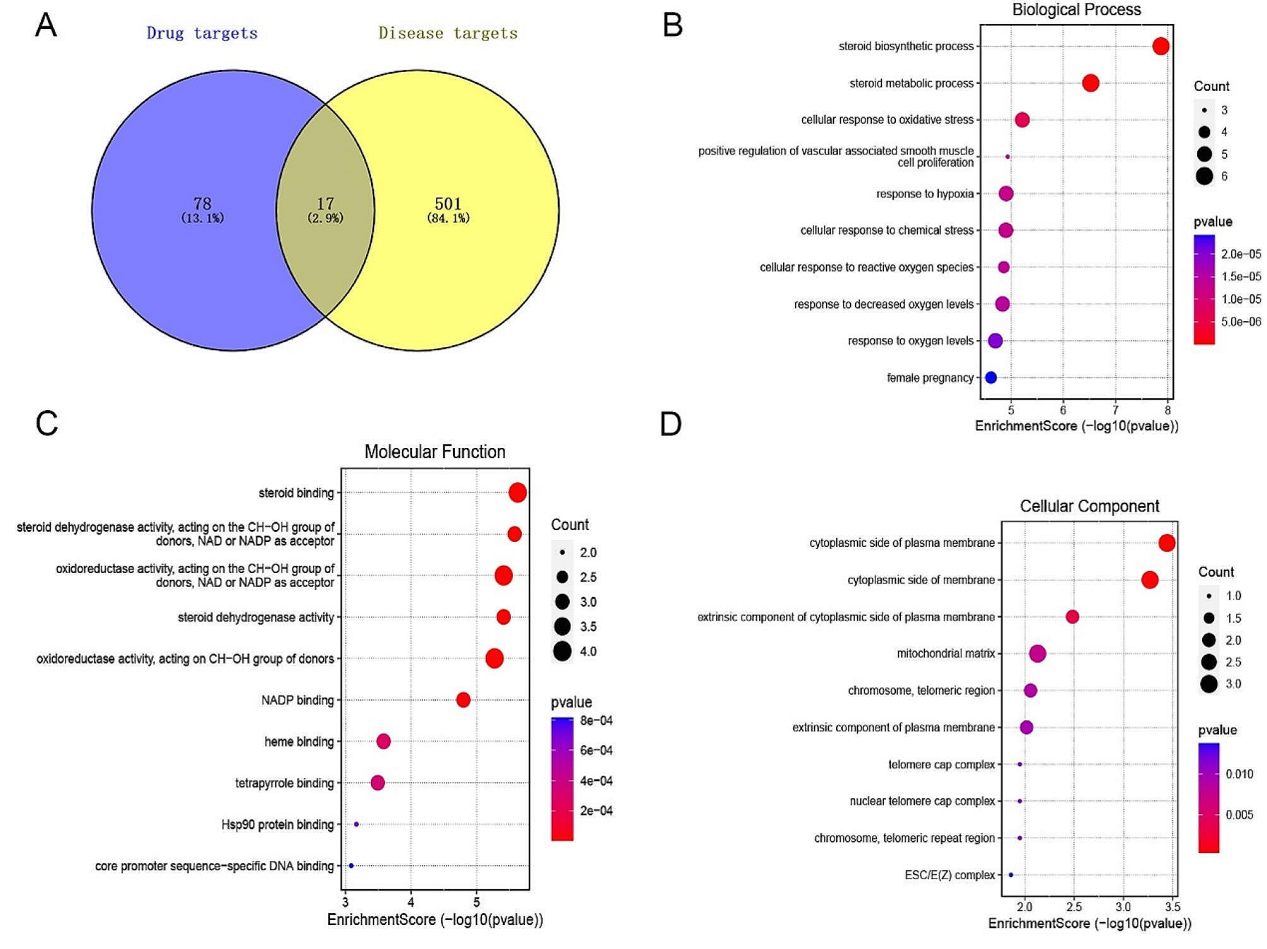
Bioinformatic analysis of astaxanthin and GIONFH. (A) Cross-gene targets between drug (Astaxanthin) and disease (GIONFH). (B) Biological Process (BP) terms for cross-gene-based Gene Ontology (GO) enrichment analysis. (C) Molecular function (MF) terms for cross-gene-based Gene Ontology (GO) enrichment analysis. (D) Cellular component terms for cross-gene-based Gene Ontology (GO) enrichment analysis

The chemical structural formula of AST was mapped by SwissTarge prediction database and a total of 78 targets were obtained (Fig. 1A). A total of 501 GIONFH disease targets were obtained from the GeneCard database (Fig. 1A). Crossing the resulting drug targets with GIONFH disease targets resulted in 17 targets for AST treatment of GIONFH (Fig. 1A). GO enrichment analysis showed that AST was involved in several biological processes (BP) of GIONFH, including steroid biosynthesis, steroid metabolism, and cellular response to oxidative stress (Fig. 1B). GO molecular function (MF) analysis showed that these target genes were closely related to steroid binding, steroid dehydrogenase activity, and oxidoreductase activity. (Fig. 1C). The cellular component (CC) term of GO analysis indicated that the target genes for crossover were enriched on the cytosolic side of the plasma membrane (Fig. 1D).

### Molecular docking and dynamic simulation between AST and Keap1-Nrf2

**Fig. 2:**
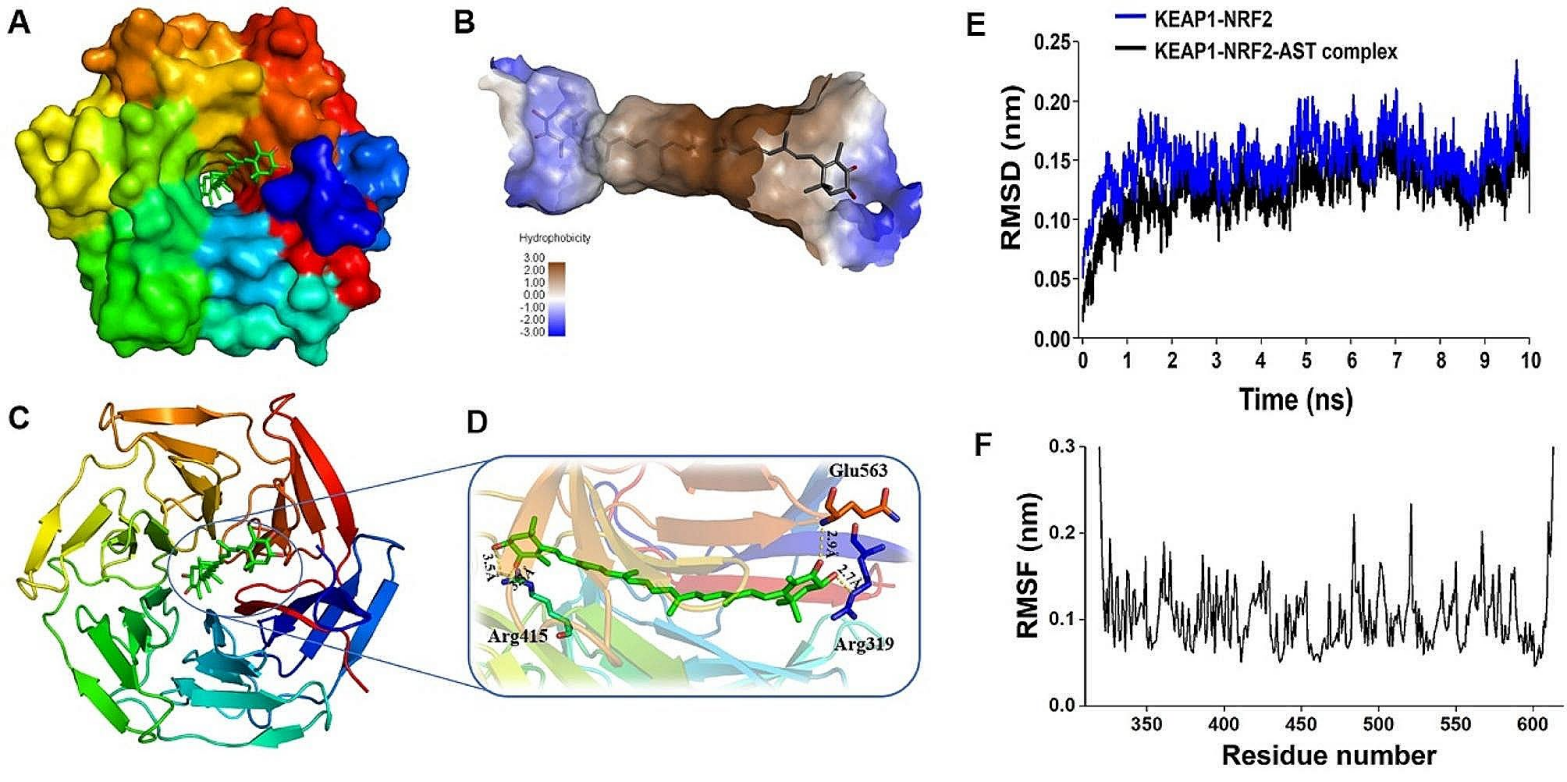
Molecular docking and dynamic simulation of AST with Keap1-Nrf2. (A) Molecular structure of Astaxanthin (AST). (B) Cartoon image of KEAP1 in complex with the astaxanthin molecule in the protein center. (C) Keap1-Nrf2 (PDB ID: 6LRZ) β-propeller domain is shown from a top view with six blades folded into pseudo-six-fold symmetry. (D) Cartoon representation of intermolecular interactions between KEAP1-Nrf2 and AST. AST produces hydrophilic interactions with binding site residues at the top and bottom sites. Important components in establishing hydrophilic interactions are Arg 319, Arg 415, and Gln 563. (E), Keap-Nrf2 and Keap-Nrf2-tax complexes yielded RMSD of 10 ns in molecular dynamics simulations. (F) Following docking, the local migration ability of the protein was analyzed by RMSF of Keap1-Nrf2 residues

The molecular structure of AST is shown in Fig. 2A. Keap1 (PDB ID: 6LRZ) β-propeller domain is shown from the top view. The propeller domain contains six blades folded into pseudo-six-fold symmetry (Fig. 2C). Keap1-Nrf2 (PDB ID: 5WFV) binding to an AST molecule located in the center of the protein is shown by cartoon representation (Fig. 2B). Docking studies for AST (AST, protein data bank (PDB) ID: AXT) and Keap1-Nrf2 (PDB ID: 5WFV). Molecular docking results of the Keap1-Nrf2-AST complex showed that molecular docking analysis of the KEAP1-NRF2-AST complex showed that AST produced significant interactions at two binding sites, one at the bottom of the nrf2 binding site and one at the top of the loop (Fig. 2C). Hydrogen bonds act on both ends of the AST molecule with residues Arg 319, Arg 415, and Gln 563 (Fig. 2D). At the bottom end, there is a hydrogen bond between the alb-ionone loop at the end and the Arg415 residue. Another b-ionone loop interacts directly with residues Arg319 and gln563 located on top of KEAP1.To mimic the binding stability of protein ligand complexes under dynamic conditions, Keap1-Nrf2 and Keap1-Nrf2-AST complexes were simulated for 10 ns. The trajectory stability of the noncoordinating target Keap1- nrf2 and target Keap1- nrf2- AST complexes was examined by the root mean square deviation (RMSD) of the Keap1 backbone (Fig. 2E).Our results show that RMSD reaches equilibrium after 6 ns of simulation and fluctuates around the mean (Fig. 2E).After 8 ns, the stable RMSD value of Keap1-Nrf2-AST complex was lower than that of the original Keap1-Nrf2 protein (Fig. 2E).The time-averaged root mean square fluctuations of protein residues were used to analyze the local mobility of proteins. The root mean square fluctuation was less than 0.1 nm and the critical amino acids were consistent with the process shown in Fig. 2F. The above analysis suggests that the Keap1-Nrf2-AST complex has a relatively stable conformation and AST may have a competitive effect on the Keap1-Nrf2 peptide target.

### Effect of AST and Dex on osteoblast viability

**Fig. 3:**
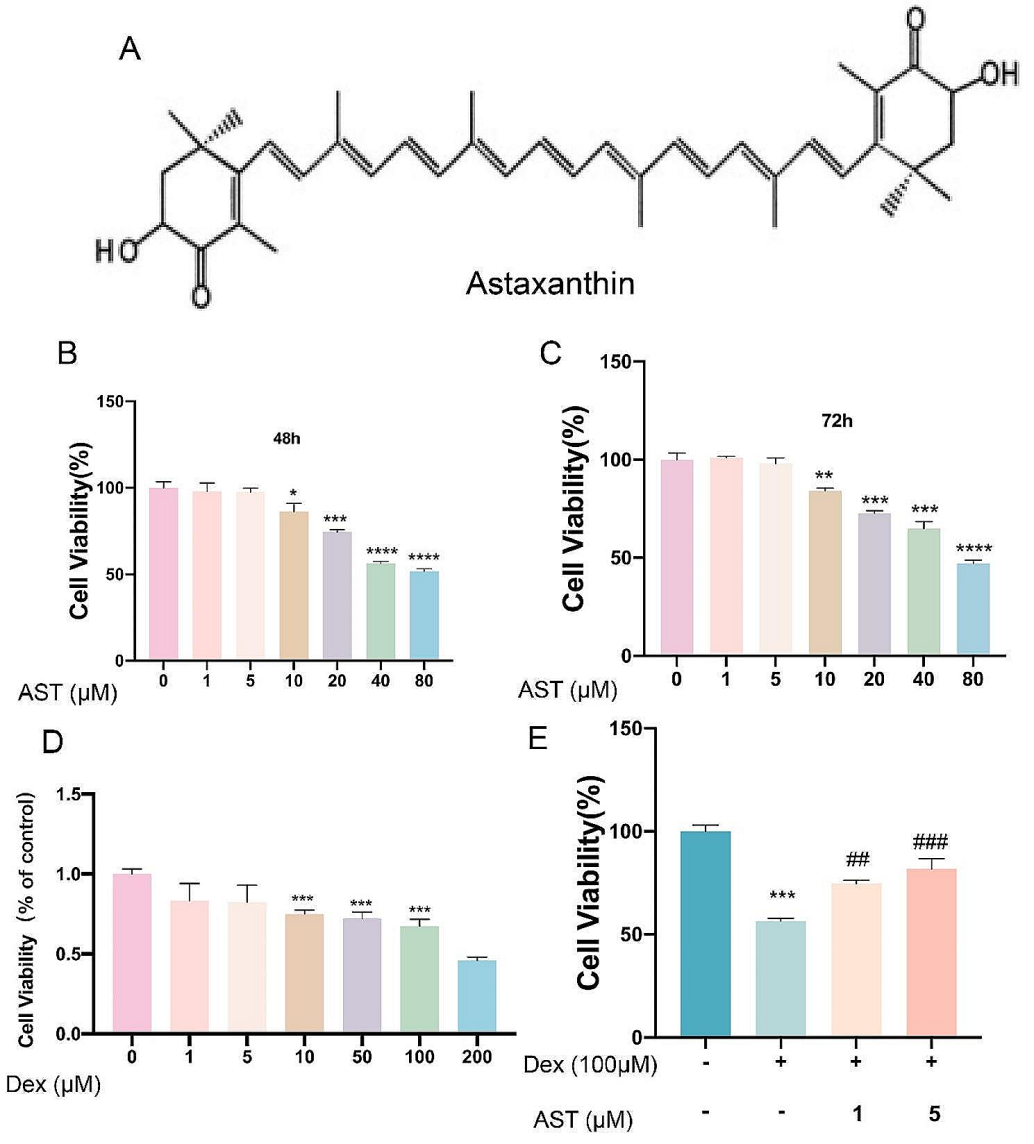
Effect of Astaxanthin and Dexamethasone on osteoblasts viability. (A) Astaxanthin chemical structure. (B-C) Evaluation of osteoblasts viability using CCK-8 assay after exposure to differing concentrations of Astaxanthin for 48 h and 72 h. (D) Evaluation of osteoblasts viability using CCK-8 assay after exposure to differing concentrations of Dexamethasone for 24 h (). (E) Percentage of viable cells pretreated with Dex with/without different concentrations of AST (*p < 0.05; **p < 0.01; ***p < 0.001 versus control group; ^##^*p* < 0.01; ^###^*p* < 0.001versus Dex group). These studies were performed at least three biological replicates

AST is a major marine carotenoid pigment produced by marine animals and has a molecular formula of C40H52O4, comprising 40 carbon atoms (Fig. 3A). Additionally, AST’s conjugated double bonds enable it to serve as a potent antioxidant via electron interaction and reaction with free radicals. In this investigation, we administered AST at varying doses to primary osteoblasts, with 5 µM being the maximum non-toxic quantity (Fig. 3BC).

To learn how Dex affected bone cells’ vitality, bone cells were cultured in DMEMs with various Dex concentrations. The findings demonstrated that Dex has a concentration-dependent adverse effect on the cellular viability of bone cells. It has a connection (Fig. 3D). The effect on bone cell viability increases with increasing Dex concentration (Fig. 3D). A 100µM concentration of Dex is chosen to further investigate its effects on bone cell oxidative stress and cell death based on the DEX concentrations utilized in other studies and our experimental findings (Fig. 3E). Dex-treated cells’ reduced vitality was restored by AST in a dose-dependent way (Fig. 3D). As per these results, subsequent experiments were performed using 1 and 5 µM AST.

### AST can reduce dex-induced mitochondrial membrane dysfunction and oxidative stress

**Fig. 4:**
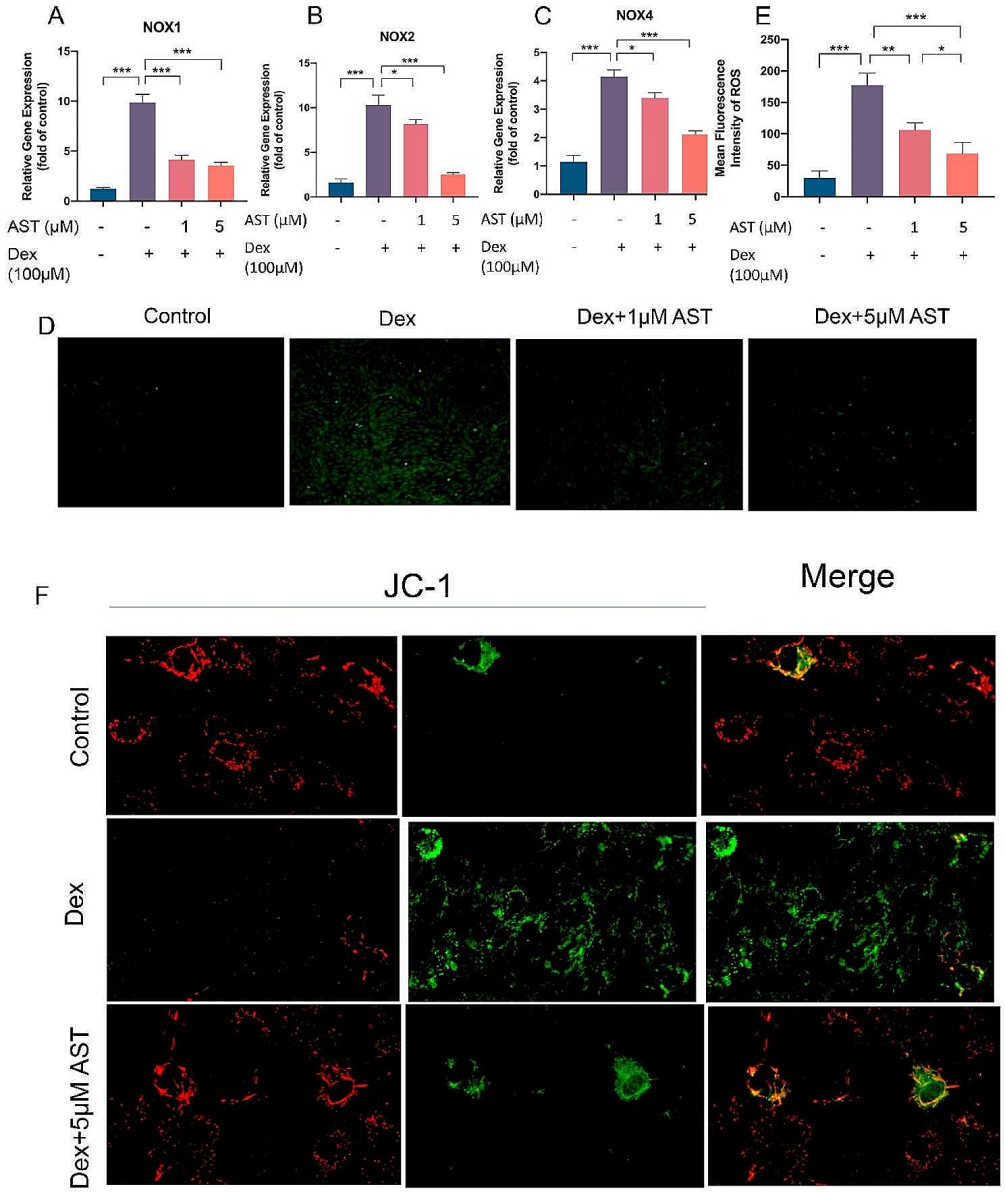
AST neutralized Dex-induced oxidative stress and mitochondrial dysfunction in osteoblasts. (A-C) The osteoblasts were treated with 100 µM DEX, 1 µM AST and 5µM AST respectively in different groups, and the gene expressions of NOX1, NOX2, and NOX4 were analyzed by qRT-PCR. (D) The mean fluorescence intensity of ROS was determined after different treatments. (E) Reactive oxygen species (ROS) assay was performed to test the level of oxidative stress under different treatments (scale bar, 100 μm). (F) Representative images showing JC-1 intensities in the differentially treated osteoblasts. (*n* = 3, mean ± SD; *p < 0.05; **p < 0.01; ***p < 0.001). These studies were performed at least three biological replicates

Four members of the NADPH oxidase (Nox) enzyme family (Nox1, Nox2, Nox3, and Nox4) are important sources of ROS in many tissues. qPCR showed that the Dex group had higher NOX1, NOX2, and NOX4 levels than the control group, which was reversed by AST pre-treatment (Fig. 4A-C). High OxS levels and osteoblast mitochondrial dysfunction lead to decreased osteogenic potential, leading to the development of GIONFH. As a result, we examined AST’s impact on ROS production and mitochondrial dysfunction in osteoblasts treated with Dex. Dex exposure increased ROS accumulation compared to untreated cells, which was neutralized by AST pre-treatment. Moreover, AST concentration was proportional to the ability to neutralize ROS, with higher AST concentrations leading to stronger neutralization of ROS (Fig. 4D-E). The JC-1 was used for estimating MMP. JC-1 is a dual fluorescent dye with good permeability to cellular and mitochondrial membranes. The decrease in the red-green fluorescence ratio indicated that mitochondria depolarised significantly less MMP in Dex-treated cells compared to the control cells, whereas both returned to near physiological levels following AST treatment (Fig. 4F).

### AST reduces DEX-induced osteoblast apoptosis

**Fig. 5:**
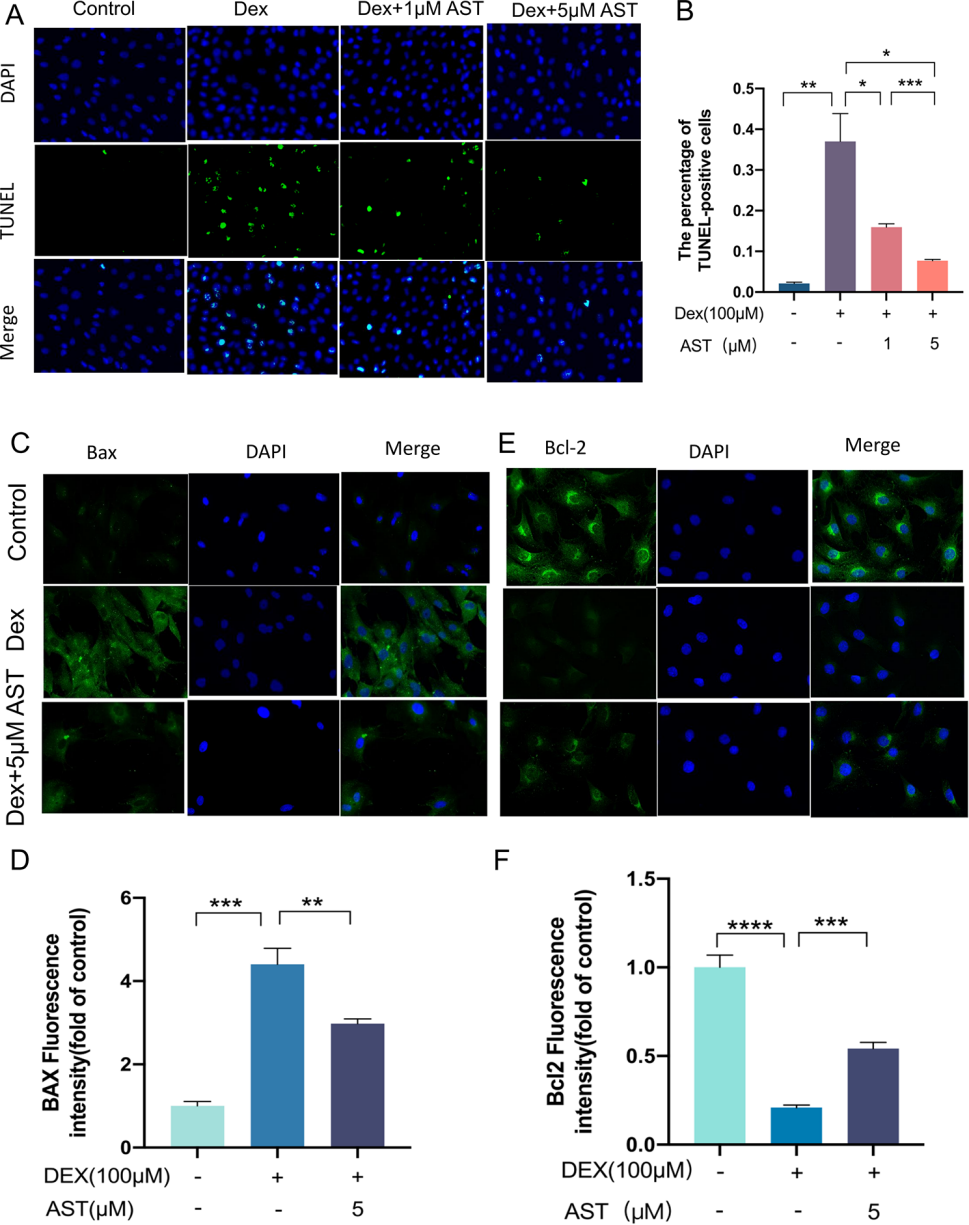
Astaxanthin attenuated DEX-induced osteoblast apoptosis. (A) The osteoblasts were treated with 100 µM DEX, 1µM AST and 5 µM AST respectively in different groups, and TUNEL staining was performed to test apoptotic rate (scale bar, 200 μm). (B) Quantitative analysis of the positively TUNEL-stained osteoblasts ratio in (A). (C-F) Representative immunofluorescence images of Bax and Bcl-2 in osteoblast in different groups and quantitative analysis of fluorescence intensity in each group. (scale bar, 100 μm) (*n* = 3, mean ± SD; *p < 0.05; **p < 0.01; ***p < 0.001). These studies were performed at least three biological replicates

Dex treatment significantly increased the number of TUNEL-positive apoptotic osteoblasts, which was alleviated by AST (Fig. 5A-B). Immunofluorescence results showed that Bax was not expressed in the control group, but increased in the cytoplasm of the Dex group. However, the intensity of Bax luminescence was greatly reduced when osteoblasts were treated with AST and Dex simultaneously (Fig. 5C-D). Furthermore, Bcl-2 expression in the control group was considerably higher, while Bcl-2 luminescence intensity was considerably lower in Dex-treated osteoblasts, however, it was higher when treated with both AST and Dex than Dex alone (Fig. 5E-F).

### AST reduces osteoblast apoptosis through the mitochondrial pathway and counteracts the DEX-induced suppression of the Nrf2 pathway

**Fig. 6:**
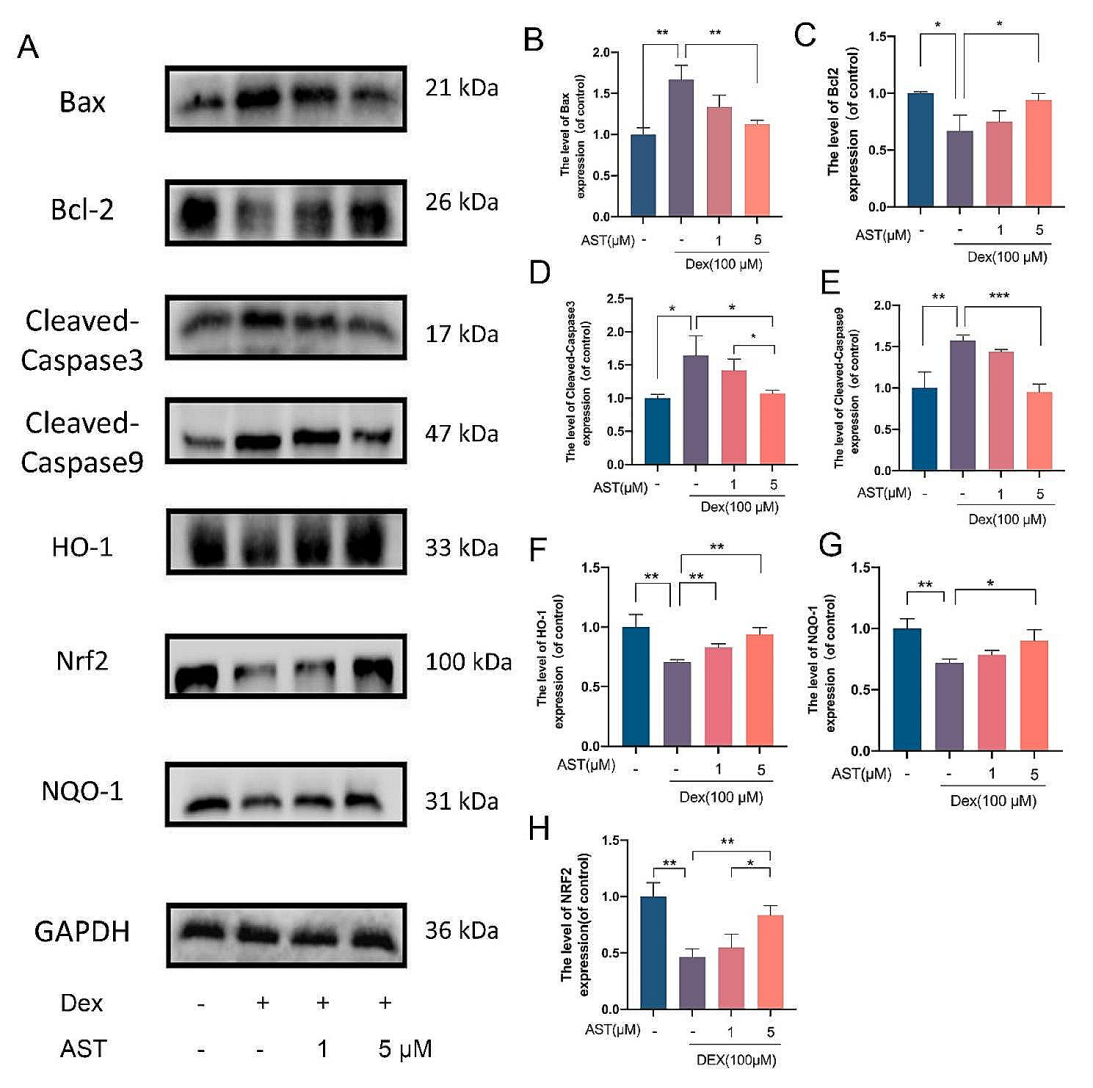
Astaxanthin attenuated osteoblast apoptosis via the mitochondrial pathway and reversed DEX-induced inhibition of the Nrf2 pathway. (A-H) The osteoblasts were treated with 100 µM DEX, 1 µM AST and 5µM AST respectively in different groups, and the protein expressions of Cleaved-Caspase9, Cleaved-Caspase3, Bax, Bcl-2, Nrf2, NQO-1, HO-1 were analyzed by Western blotting. (*n* = 3, mean ± SD; *p < 0.05; **p < 0.01; ***p < 0.001). These studies were performed at least three biological replicates

Similar results for Bax and Bcl-2 were observed using immunofluorescence as well as western blotting, suggesting that AST may inhibit Dex-induced apoptosis by decreasing Bax levels and raising Bcl-2 levels (Fig. 6A-C). Furthermore, caspase-9 and caspase-3, the key effectors of mitochondrial apoptosis, were examined by western blotting. western blotting showed consistent upregulation of cleaved caspase-9, and cleaved caspase-3 in Dex-treated cells, with normalization of these proteins following AST administration (Fig. 6A, D-E). More notably, Nrf2 pathway-related proteins, including HO-1, NRF2 and NQO-1, were decreased in Dex group, which was reversed by AST treatment (Fig. 6A, F-H), suggests that Nrf2 signal pathway is involved in AST decreasing dex-induced mechanisms of bone cell death. Overall, our findings suggest that AST can protect against Dex-induced osteoblast damage.

### AST restores differentiation and mineralization of Dex-treated osteoblasts

**Fig. 7:**
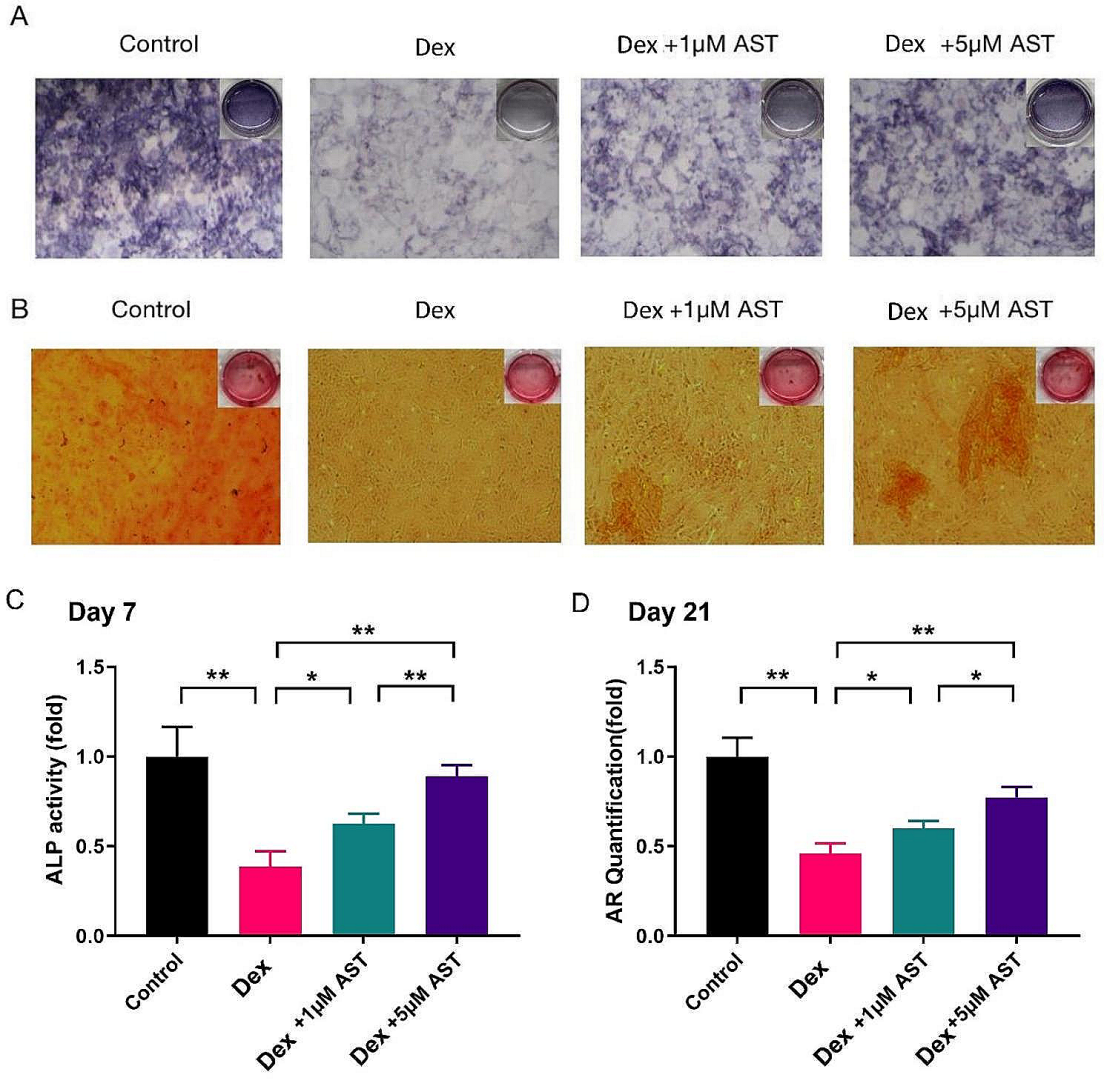
Representative images (A and C) showed ALP expression and ALP activity in osteoblasts on the 7th day by ALP staining. (B and D) The mineralization degree of osteoblasts was detected and quantified by ARS staining on the 21st day. (scale bar, 100 μm) (*n* = 3, mean ± SD; *p < 0.05; **p < 0.01; ***p < 0.001). These studies were performed at least three biological replicates

In osteoblasts, OxS and mitochondrial dysfunction suppress osteogenic function [23, 24]. Therefore, in cells treated with Dex, we evaluated how AST affected early differentiation and mineralization. After seven days in culture, Dex dramatically reduced osteogenic differentiation as shown by ALP activity, and on day 21, it attenuated the formation of calcium nodules. However, in Dex-treated osteoblasts, combined treatment with AST restored ALP activity and mineralization (Fig. 7A-D). Overall, AST protected osteoblasts from the inhibitory effects of Dex on osteogenic differentiation and the formation of calcium nodules.

### AST protects osteogenic cells from Dex-induced apoptosis through the mitochondrial pathway by activating the Keap1/Nrf2 signaling pathway

**Fig. 8:**
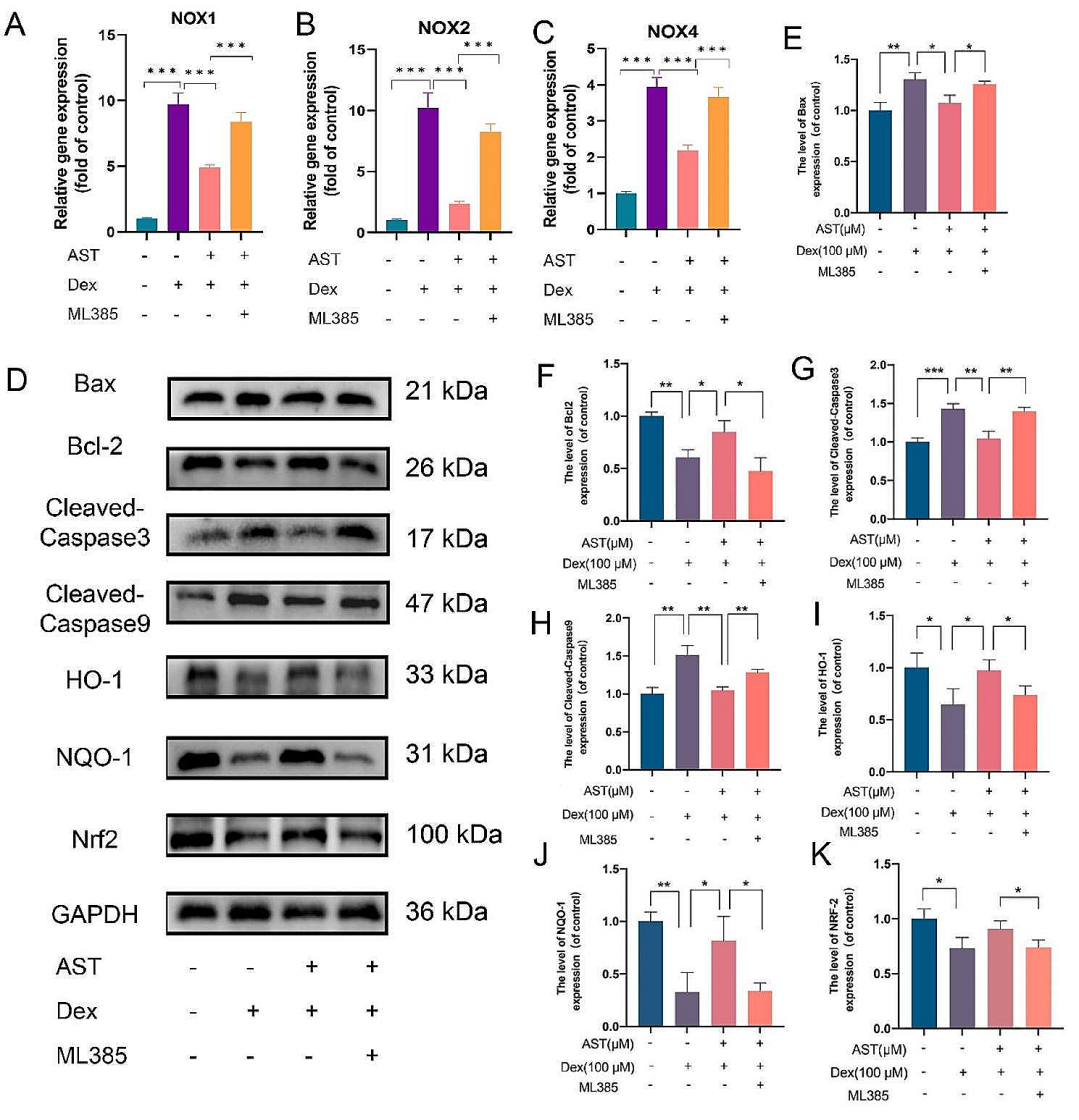
AST protects osteogenic cells from Dex-induced apoptosis through the mitochondrial pathway by activating the Keap1/Nrf2 signaling pathway. (A-C) The osteoblasts were treated with 100 µM Dex, 5µM AST and 20µM ML385 respectively in different groups. And the gene expressions of NOX1, NOX2, and NOX4 were analyzed by qRT-PCR. (E-H) The protein expressions of Cleaved-Caspase9, Cleaved-Caspase3, Bax, Bcl2 were analyzed by Western blotting. (I-K) The protein expressions of Nrf2, NQO1, HO1 were analyzed by Western blotting. (*n* = 3, mean ± SD; *p < 0.05; **p < 0.01; ***p < 0.001). These studies were performed at least three biological replicates

In a previous study, AST increased Nrf2 nuclear translocation and enhanced the expression of NAD(P)H, NQO1, superoxide dismutase, and HO-1 in a high glucose-induced diabetic nephropathy injury model [25], indicating that the Nrf2 pathway may mediate AST’s protective effect [26]. We employed ML385 to suppress Nrf2 expression in osteoblasts to further confirm the protective impact of AST on these cells. In this part, ML385 reversed the inhibitory effects of AST on the expressions of NOX1, NOX2 and NOX4 in Dex-induced cells (Fig. 8A-C). Furthermore, western blotting demonstrated that ML385 partly reversed the regulatory effects on apoptosis-related proteins, including, Bax, Bcl-2, cleaved caspase-3 and cleaved caspase-9 (Fig. 8E-H). AST treatment reversed Dex’s inhibition of HO-1, NQO-1 and Nrf2 nuclear transfer levels (Fig. 8D, I-K). However, ML385 dramatically reversed the effect caused by AST treatment (Fig. 8D, I-K). These findings support the notion that AST protects osteogenic cells from Dex-induced apoptosis through the mitochondrial pathway by activating the Keap1/Nrf2 signaling pathway.

### AST rescued MPS-initiated dysfunction in poor bone quality of the femoral head in rats

**Fig. 9:**
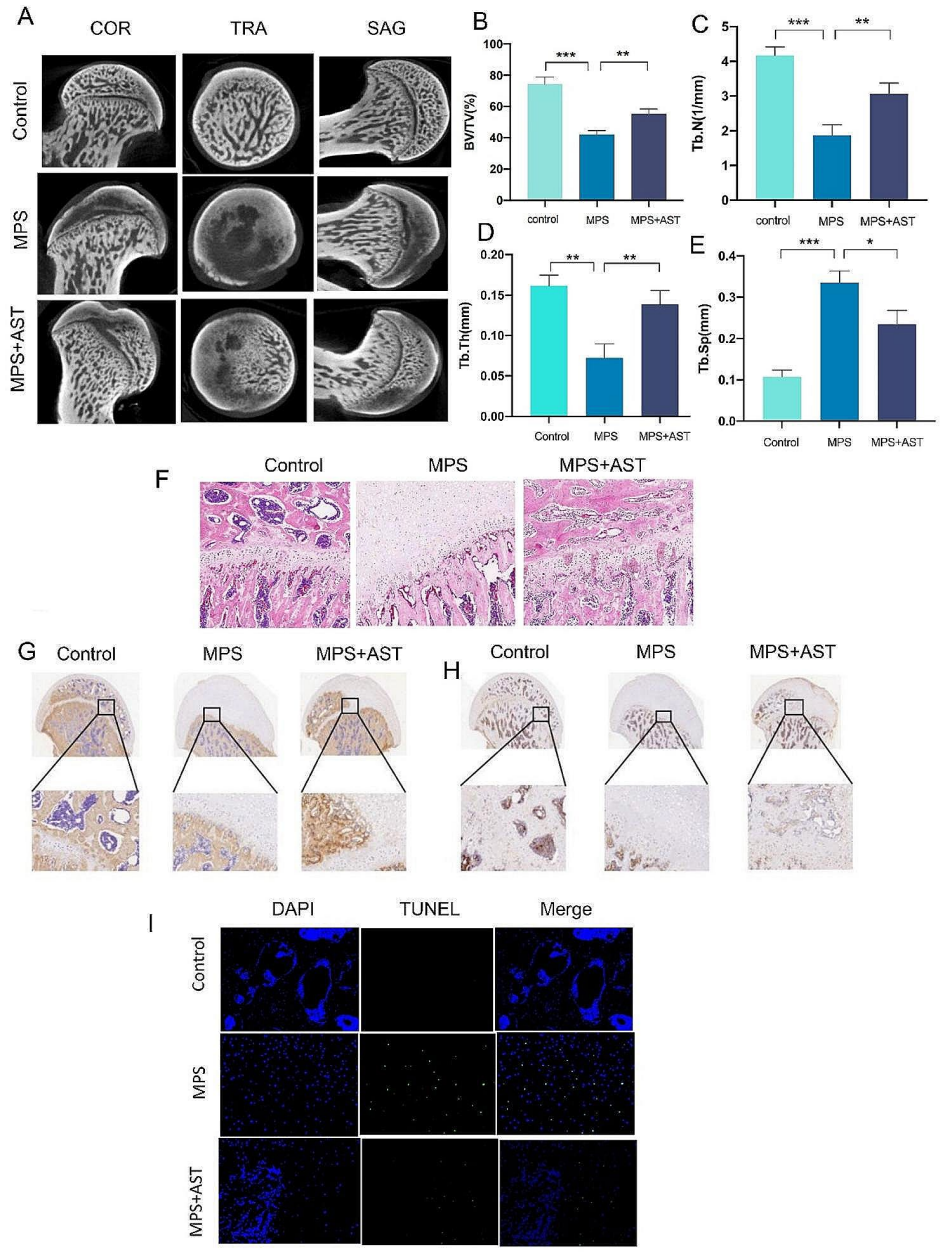
Astaxanthin rescued MPS-initiated dysfunction in poor bone quality of the femoral head in a rat model. (A) Three plane images including coronal section (COR), transverse section (TRA) and sagittal section (SAG) of the femoral head were reconstructed in the different groups. (B-E) Quantitative analysis of micro-CT results. Bone volume per tissue volume (BV/TV), Trabecular number (Tb.N), Trabecular thickness (Tb.Th) and Trabecular separation (Tb.Sp) were analyzed in the different groups. (F) HE staining of the femoral head in the different groups (scale bar, 200 μm). (G) IHC images showing COL-I staining of the femoral head in the different groups (scale bar, 200µ m). (H) IHC images showing HO-1 staining of the femoral head in the different groups (scale bar, 200 μm). (I) TUNEL staining images showing apoptotic cells in the different groups (scale bar, 200 μm). (*p < 0.05; **p < 0.01; ***p < 0.001)

The GIONFH rat model was employed in this work to test our hypothesis further. Micro-CT scanning was used to assess the microstructures of the distal femur, and several parameters such as Tb.N, Tb.Sp, BV/TV, and Tb.Th were calculated for each group (Fig. 9A-E). AST for eight weeks had significantly improved BV/TV and Tb.N and decreased Tb.sp compared to MPS-treated rats. Histological examinations of distal femur sections from the GIONFH model treated with AST demonstrated an increase in trabeculae compared to MPS-treated rats (Fig. 9F). Furthermore, IHC revealed that COL-1 and HO-1 expression was higher in MPS + AST (MPS and AST treatment) groups (Fig. 9G, H). The results of this study provide strong evidence that AST exerts a potential therapeutic impact on GIONFH by improving poor bone quality of the femoral head.

### AST rescued MPS-initiated dysfunction in bone metabolism- and angiogenesis- related markers of the femoral head in rats

**Fig. 10:**
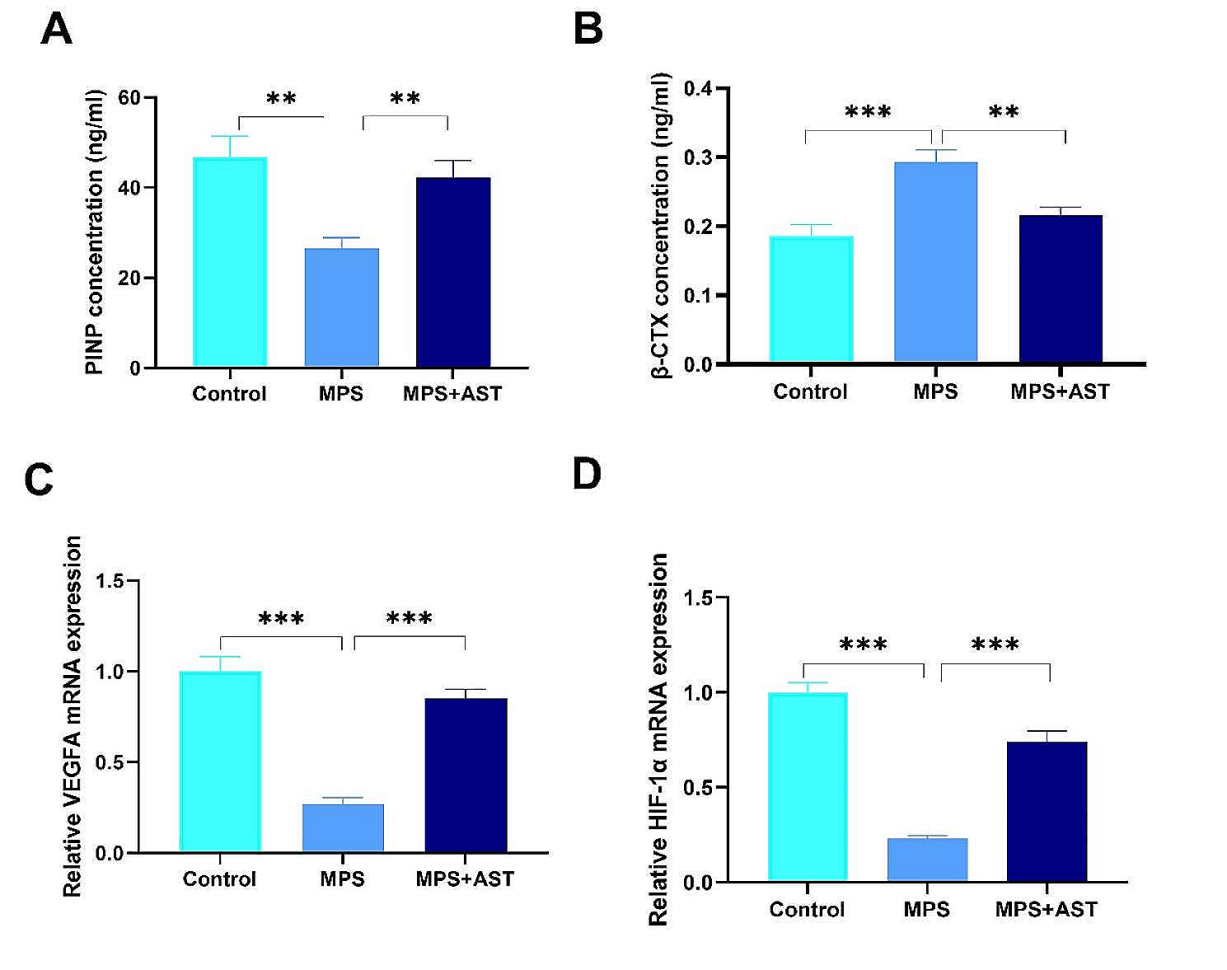
Astaxanthin rescued MPS-initiated dysfunction in bone metabolism- and angiogenesis- related markers of the femoral head in a rat model. (A-B) Serum markers of bone formation (PINP) and bone resorption (β-CTX) measured by using ELISA assay. (C-D) Relative mRNA levels of VEGF and HIF-1α assessed by qRT-PCR. (^**^p < 0.01; ^***^p < 0.001)

In accordance with the findings regarding poor bone quality mentioned above, the level of serum PINP (a bone formation marker) was decreased by MPS, while the level of serum β-CTX (a bone resorption marker) was increased by MPS; however, AST reversed these effects (Fig. 10AB). Furthermore, the angiogenesis-related markers of VEGFA and HIF-1α levels were lower in the MPS group than those in the control group; however, AST also reversed these effects (Fig. 10CD).

## Discussion

In this study, for the first time, we demonstrate that AST protects rat osteoblasts against Dex-caused osteogenic damage, OxS, and mitochondrial dysfunction. Dex has been shown to decrease Nrf2 pathway protein expression by increasing ROS generation. As a critical transcription factor, Nrf2 restores intracellular redox homeostasis by binding to ARE, including the downstream transcriptional effectors NQO-1 and HO-1, thereby exerting cytoprotective effects [27]. Based on network pharmacology and molecular docking techniques, we determined that AST could modulate GIONFH oxidative stress and bone regeneration. We hypothesized that AST could directly interact with Keap1, which is a major repressor of Nrf2. From the molecular docking map, AST can accurately bind to the conjugation site of Keap1. Their dynamic results showed that AST competes for keap1 target peptide labeling. AST activates the antioxidant Nrf2 pathway, significantly raising NQO-1 and HO-1 expression as well as that of the downstream effectors and, as a result, attenuating ROS formation and lowering mitochondrial superoxide levels in osteoblasts. This result suggests that OxS is responsible for alterations in osteoblast differentiation direction. Furthermore, AST increases osteoblast viability and differentiation via modulating the Nrf2 signaling pathway.

GC has been utilized extensively in the treatment of, autoimmune, rheumatic, and blood topological disorders. However, its abuse is one of the leading causes of non-traumatic ONFH, which has been observed in 5–40% of GC-treated patients [28–30]. Moreover, the risk of ONFH increases with increasing dose and treatment. The femoral head finally collapses with the progression of ONFH, leaving patients with pain and disability. By reducing osteoblast viability and differentiation, GC reduces bone growth [31]. As a synthetic GC, Dex has been shown to decrease osteoblast growth while also promoting apoptosis by inducing OxS and mitochondrial dysfunction [32, 33]. Dex treatment, according to Liu et al., increased MMP loss and ROS accumulation in MC3T3-E1 osteoblasts [34]. Notably, ROS accumulation in the mitochondria establishes a vicious cycle of OxS and mitochondrial dysfunction, eventually leading to the induction of apoptotic pathways [35]. Xtiao et al. observed that Dex promotes ER stress and mitochondrial apoptosis in osteoblasts [36]. In line with these findings, we discovered that Dex therapy caused osteoblast malfunction and apoptosis. As a result, lowering OxS and mitochondrial dysfunction in osteoblasts might be a potentially useful therapy for AST.

Mitochondria are the main organelles that play a critical role in osteoblasts as they participate in energy metabolism and calcium homeostasis and regulate cell survival, death, and cell function [25]. GC-induced bone metabolism disorders are primarily caused by mitochondrial dysfunction, which leads to cellular dysfunction [28]. Two oxygenated β-ionone-type ring systems are joined by conjugated double-bond chains (polyene chains) in the structure of AST. The hydroxyl (OH) and ketone (C = O) moieties of the oxygen atoms in the AST ionone ring give this carotenoid its strong antioxidant activity and polarity, classifying it as a member of the lutein carotenoid family [34, 37, 38]. Wu et al. [39] found that in patients with non-alcoholic fatty liver disease, AST alleviated liver injury and mitochondrial dysfunction. In addition to decreasing muscle atrophy [40], AST also protects mitochondrial functional integrity by reducing mitochondrial OxS and mitochondria-mediated apoptosis. When mitochondria are compromised, a pore opens in the mitochondrial membrane, and MMP is lost. The fluorescent dye generates a bright red light when it forms highly charged J aggregates in a healthy mitochondrial matrix. However, in affected mitochondria with reduced membrane potential, the dye fluoresces green as a monomer. The loss of MMPs indicates mitochondrial dysfunction and is an early marker of apoptosis. Therefore, the easily detectable change in color of JC-1 fluorescence from red to green is seen as evidence of early, Dex-induced apoptosis. MMP was evaluated in our study using JC-1 dye, which demonstrated that AST reduced Dex-induced mitochondrial membrane damage in osteoblasts in vitro. This suggests that AST ameliorates Dex-induced osteoblast dysfunction by stabilizing MMPs. Additionally, in Dex-treated cells, AST lowered the high apoptotic rate, downregulated apoptotic protein levels, including cleaved caspase-3, Bax, and cleaved caspase-9, and upregulated Bcl-2. These findings imply that AST may inhibit osteoblast apoptosis via the mechanism of lowering OxS levels.

**Fig. 11:**
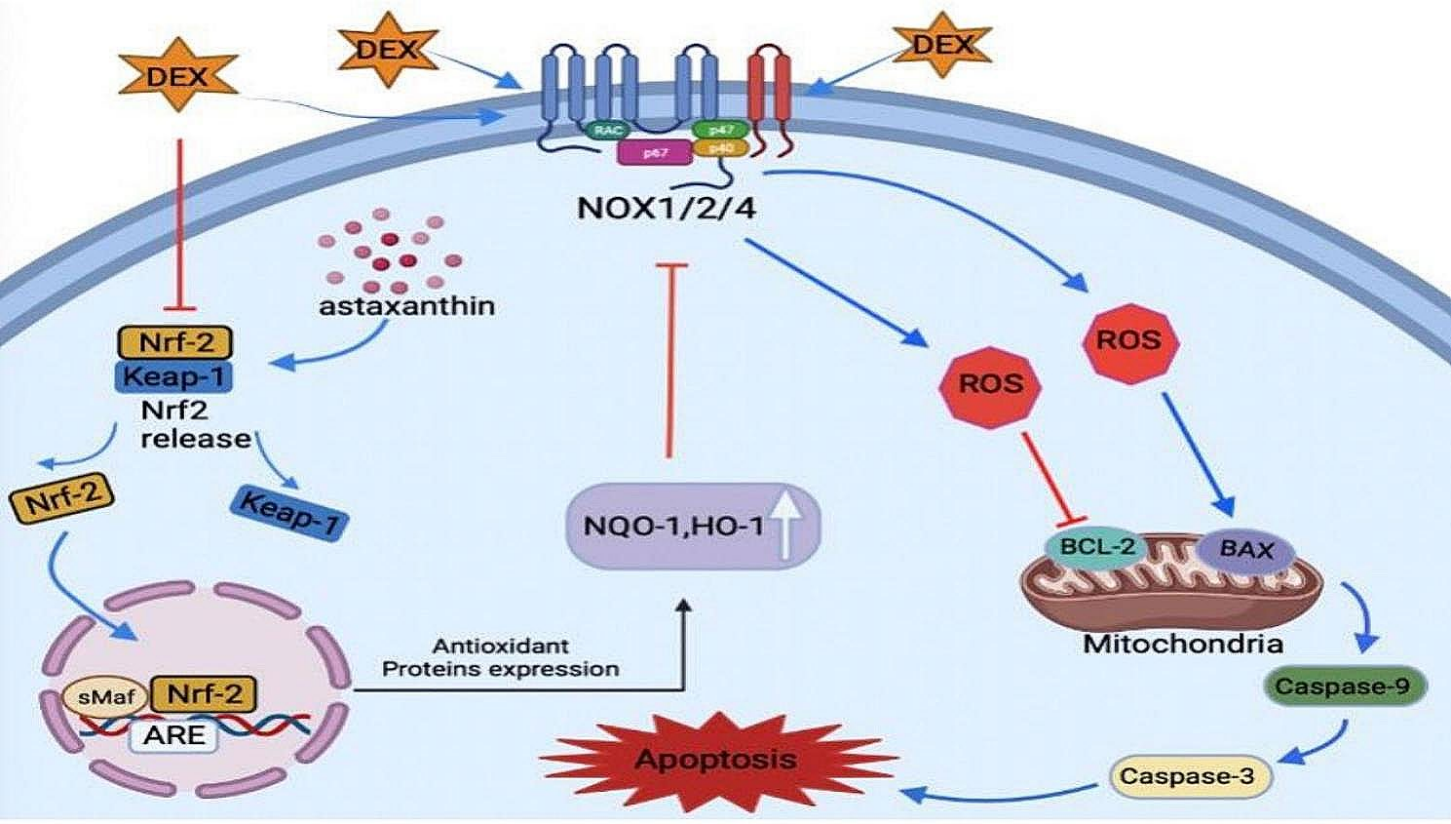
A schematic diagram illustrating the role of AST in protecting against oxidative stress and apoptosis via the Nrf2 signaling pathway

Additionally, the subchondral bone region’s bone structure in the model group experienced severe destruction, the nearby bone marrow cells were necrotic, and there were numerous hollow spaces could be seen. AST is essential in the regulation of bone growth and resorption. The effect of AST on GIONFH in rats was investigated utilizing micro-CT and histology in the current study. In rats, AST was observed to improve MPS-induced bone mass in the femoral head, increase the number of trabeculae, and decrease trabeculae separation. These findings in vivo are in accordance with the change in the content of serum bone metabolism-related markers (PINP and β-CTX). VEGF is an important downstream target gene for HIF-1α, and VEGFA as the most studied growth factor of the VEGF family facilitates angiogenesis and enhances vascular permeability. A recent study reported that the repair response in MPS-induced ANFH might be associated with the Akt/HIF‐1α/VEGF pathway [41]. Furthermore, in this study, AST also rescued MPS-initiated dysfunction in angiogenesis- related markers (HIF‐1α/VEGFA) of the femoral head in rats. AST improved the viability of osteogenic cells in Dex-induced GIONFH in vitro. Dex induction also inhibited osteoblast differentiation, whereas AST enhanced Dex-induced osteoblast differentiation, as evidenced by enhanced ALP activity and the increased number of calcified nodules. As a result of these findings, AST may stimulate bone formation by increasing osteoblast viability and differentiation, consequently reducing MPS-induced ONFH. This study concludes that AST protects rat osteoblasts from Dex-induced OxS, mitochondrial dysfunction, and osteogenic injury for the first time. Mechanistically, AST activated the antioxidant Nrf2 pathway and subsequently decreased ROS accumulation and mitochondrial superoxide levels in osteoblasts. In male rats, AST reduced skeletal loss and increased Nrf2 expression in the GIONFH model in vivo. Astaxanthin-Mediated Nrf2 Activation Ameliorates Glucocorticoid-Induced Oxidative Stress and Mitochondrial Dysfunction and Impaired Bone Formation of glucocorticoid-Induced Osteonecrosis of The Femoral Head in RatsThe fact that we did not compare the effects of AST with those of multiple clinical medications used to treat individuals with GIONFH, however, poses a potential drawback to the research we conducted. Therefore, further research is required to corroborate this. Despite these drawbacks, our research reveals that OxS and mitochondrial dysfunction brought on by Dex are harmful to the development of osteoblasts and the function of the mineralized tissues. Additionally, these findings imply that AST supplementation can dramatically enhance clinical outcomes in GIONFH patients. In conclusion, AST-mediated Nrf2 activation ameliorates glucocorticoid-induced oxidative stress and mitochondrial dysfunction and impaired bone formation of glucocorticoid-induced osteonecrosis of the femoral head in rats (Fig. 11).

Considering that male gender, longer symptom duration before treatment, higher VAS scores, and lower HHS scores are negative prognostic factors following treatment for osteonecrosis of the femoral head [42], future research could investigate whether AST supplementation influences these prognostic factors and improves treatment outcomes in patients with ONFH. Furthermore, in light of a systematic review in the management of ONFH [43], while conservative management remains the initial approach, surgery becomes necessary in cases of treatment failure. Future studies could explore the potential synergistic effects of AST supplementation in conjunction with surgical interventions such as osteotomies for the management of GIONFH. Additionally, randomized clinical trials could be conducted to evaluate the comparative efficacy of AST and other clinical medications in treating GIONFH, addressing the potential limitation identified in our study. A meta-analysis compares the outcomes of core decompression (CD) with and without bone marrow-derived cell therapies (BMCTs) in preventing or delaying the necessity of total hip arthroplasty (THA) for ONFH [44]. The findings demonstrate that CD combined with BMCTs results in reduced pain and a lower rate of THA compared to CD alone, highlighting the potential benefits of adjunctive BMCTs in the management of ONFH. Future studies could explore the synergistic effects of AST supplementation in conjunction with CD and BMCTs for GIONFH. Additionally, randomized controlled trials could be conducted to evaluate the comparative efficacy of different treatment modalities, including AST, CD, and BMCTs, in improving clinical outcomes for patients with GIONFH. Besides, a meta-analysis evaluates patient clinical status, radiographic progression, and the need for total hip arthroplasty (THA) or further surgery (FS) as outcomes [45]. The findings suggest a slight superiority of other joint preserving treatments (JPT) over core decompression (CD) in terms of clinical outcome, radiographic progression, and the need for THA/FS. Further studies could explore the potential synergistic effects of AST supplementation with various joint preserving strategies, including CD and other JPT, in improving clinical outcomes for patients with ONFH. Additionally, randomized controlled trials comparing the efficacy of different therapeutic approaches, including AST supplementation, CD, and other JPT, in delaying disease progression and preventing the need for THA/FS could provide valuable insights into the optimal management of ONFH. A review includes an analysis of various operative modalities and clinical outcomes, highlighting the effectiveness of surgical techniques in improving functional outcomes of avascular necrosis of the femoral head in skeletally immature patients [46]. Future studies could explore the potential synergistic effects of AST supplementation with surgical interventions in skeletally immature patients with ONFH, considering its protective effects on osteoblast function and bone metabolism. Additionally, comparative studies evaluating the efficacy of AST supplementation versus standard surgical treatments in improving clinical outcomes for ONFH patients could provide valuable insights into the role of AST as an adjunctive therapy in the management of ONFH. All in all, by bridging the gap between basic research and clinical practice, such interdisciplinary approaches hold promise for advancing the management of ONFH and improving clinical outcomes for affected patients.

## Data Availability

The original contributions presented in the study are included in the article materials; further inquiries can be directed to the corresponding author.
